# Nanosheet-type tin oxide gas sensor array for mental stress monitoring

**DOI:** 10.1038/s41598-022-18117-8

**Published:** 2022-08-25

**Authors:** Pil Gyu Choi, Yoshitake Masuda

**Affiliations:** National Institute of Advanced Industrial Science and Technology (AIST), 2266-98 Anagahora, Shimoshidami, Moriyama, Nagoya, 463-8560 Japan

**Keywords:** Materials science, Nanoscale materials, Electrical and electronic engineering

## Abstract

Mental stress management has become significantly important because excessive and sustained mental stress can damage human health. In recent years, various biomarkers associated with mental stress have been identified. One such biomarker is allyl mercaptan. A nanosheet-type tin oxide exhibited high gas selectivity for allyl mercaptan; thus, in this study, a sensor array comprising nanosheet-type tin oxide gas sensors was fabricated to detecting allyl mercaptan. Supervised learning algorithms were use to build gas classification models based on the principal component analysis of the sensor signal responses from the sensor array. The comprehensive data provided by the classification models can be used to forecast allyl mercaptan with high accuracy.

## Introduction

The concept of stress was first introuced in 1936 as “the non-specific response of the body to any demand,” a definition extended by subsequent stress experiments^1–3^. Recent research defines stress as any event that disrupts homeostasis, causing the body to release hormones to restore homeostasis. Chronic stress is biologically associated with several disorders and health related problems. Therefore, stress management is essential in healthcare to prevent diseases and improv the quality of life.

Mental stress can be quantified by measuring the levels of different mental stress biomarkers discharged from the body. Shiseido Co. Ltd. recently identified discernible odor substances emanating from the skin during emotional tension, and one such substance was allyl mercaptan^4–6^. As a result, the detection of allyl mercaptan could be used to prevent chronic mental stress notifying a state of an initial emotional tension to the user.

Gas sensors are effective devices in the detection of odorous substances. In addition, sensor arrays combined with statistical data analysis have been considered suitable for discriminating between, detecting, and recognizing gases^7–9^. The key to good performance of sensor arrays isthe gas sensor possessing superior and unique sensing properties. As the fundamental sensing mechanism of a gas sensor ivolves the adsorption and desorption of gas molecules on the surface of the sensor material^10–13^, a material with a different surface structure can serve as a critical material in gas sensing application.

In previous studies, a gas sensor was developed using a nanosheet-type tin oxide as the gas sensor material^14,15^, which mainly exposed metastable surface structures. The sensor also exhibited superior and unique sensing properties owing to its different surface structures^16–19^.

In this study, we investigated the properties of nanosheet-type tin oxides to allyl mercaptan. We designed a gas sensor array consisting of nanosheet-type tin oxide gas sensors and conducted a gas discrimination and forecasting study through statistical forecasting models using the collected sensor signals for biomarkers, including allyl mercaptan.

## Results

**Figure 1:**
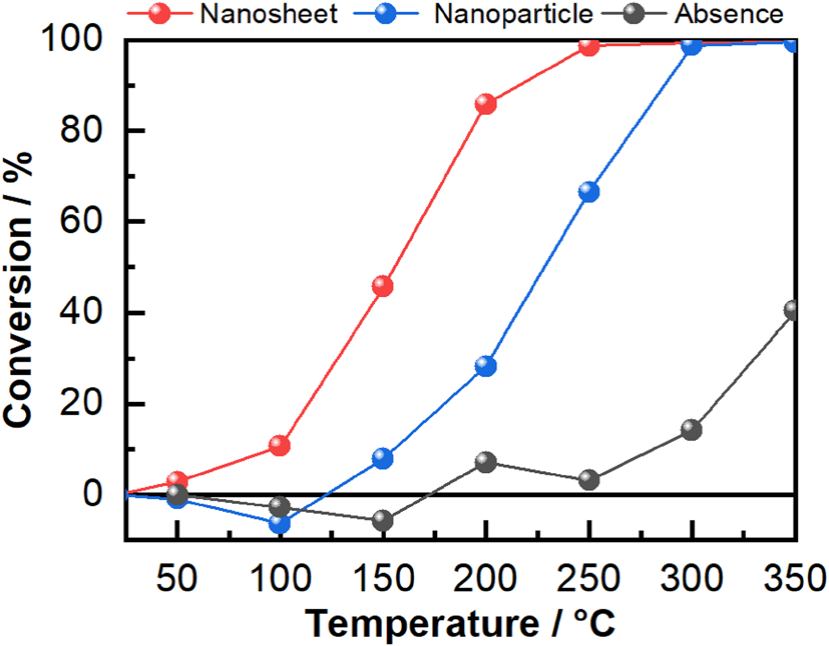
Allyl mercaptan conversion percentage (powder: 0.01 g, flow: 100 cm^3^/min, gas concentration: 20 ppm, tube diameter: 10 mm).

The catalytic oxidation activity of allyl mercaptan was investigated using nanosheet-type tin oxide, and commercial tin oxide nanoparticles were used for comparison (Fig. 1). The nanosheet-type tin oxide exhibited higher activity toward allyl mercaptan than the commercial tin oxide nanoparticles. A conversion reaction began in nanosheet-type tin oxide at approximately 100 °C, and the conversion percentage reached approximately 99% at 250 °C. In contrast, in the case of commercial tin oxide nanoparticle, the conversion reaction began to progress at approximately 150 °C, and the conversion percentage was approximately 99% at 300 °C. Allyl mercaptan reacts spontaneously with oxygen without any additives at temperatures above 300 °C. The higher activity of nanosheet-type tin oxide may be attributed to its metastable surface structure with high reactivity.

**Figure 2:**
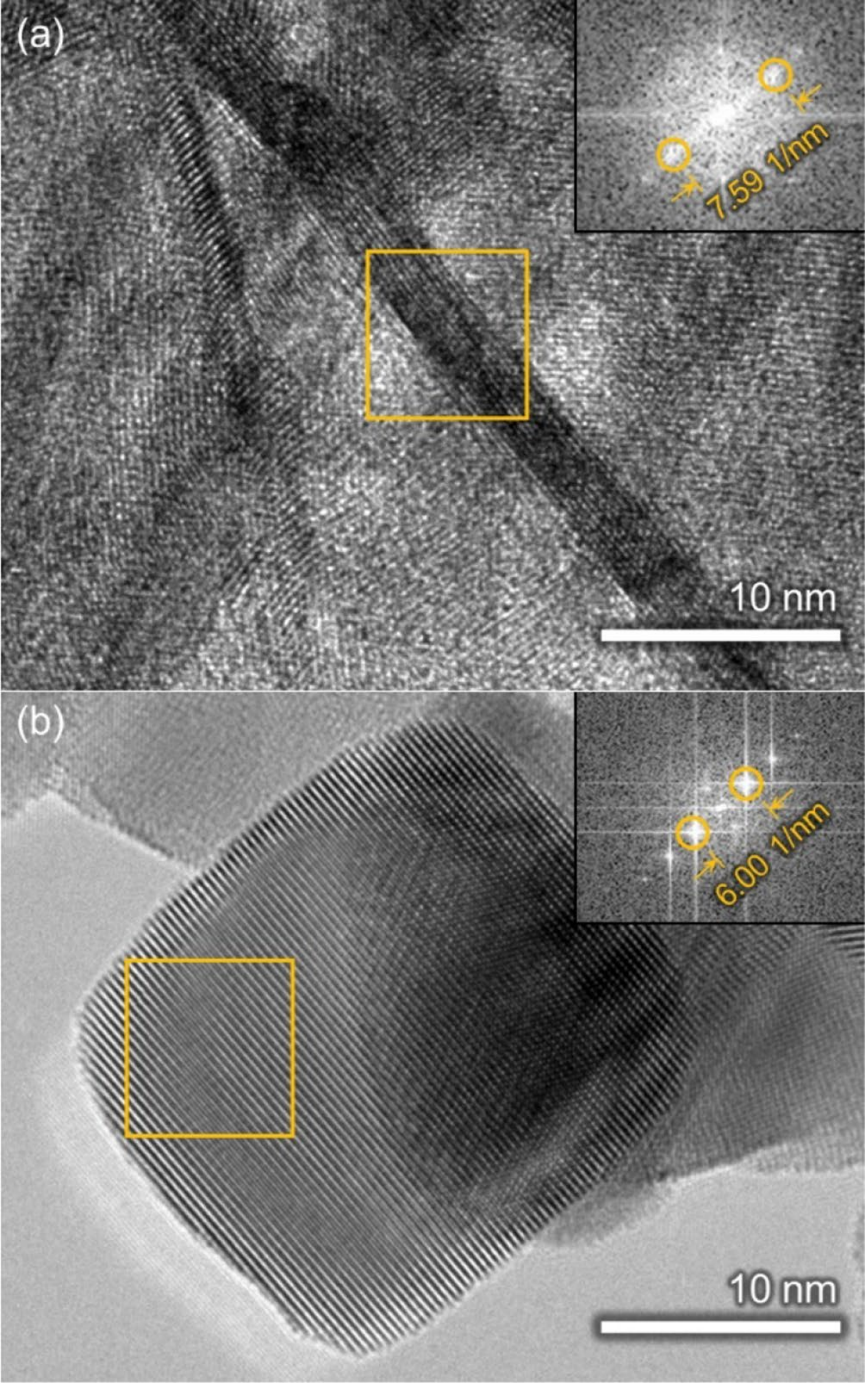
Transmission electron microscopy (TEM) images of (**a**) nanosheet-type tin oxide and (**b**) commercial tin oxide nanoparticle with corresponding fast Fourier transform (FFT) (insert).

The arrangement of the atoms was observed using transmission electron microscopy (TEM) (Fig. 2). A layer distance of d = 0.263 nm was observed in the atomic arrangement shown in the cross-section of nanosheet-type tin oxide, corresponding to the d space of SnO_2_ (101). A layer distance of d = 0.333 nm was observed in the atomic arrangement of the commercial tin oxide nanoparticles, corresponding to the d space of SnO_2_ (110). This indicates that the nanosheet-type tin oxide mainly expose the metastable SnO_2_ (101) crystal facet on the surface. In contrast, the commercial tin oxide nanoparticles mainly exposes the most stable SnO_2_ (110) on the surface.

The SnO_2_ (101) surface has been reported to expose a large amount of bridging oxygen to comparison with the SnO_2_ (110) surface, which proves favorable for redox reactions^20^. Moreover, the nanosheet-type tin oxide was considered to possess crystal defects on the surface^16,19^. Although it was difficult to determine the type of crystal defects, it may be attributed to the metastable state of the SnO_2_ (101) surface. Considering that crystal defects predominantly affect activity, it was suggested that large amount of bridging oxygen and the crystal defects attributed to the metastable state of the SnO_2_ (101) facet, further contributing to the high activity of the nanosheet-type tin oxide.

**Figure 3:**
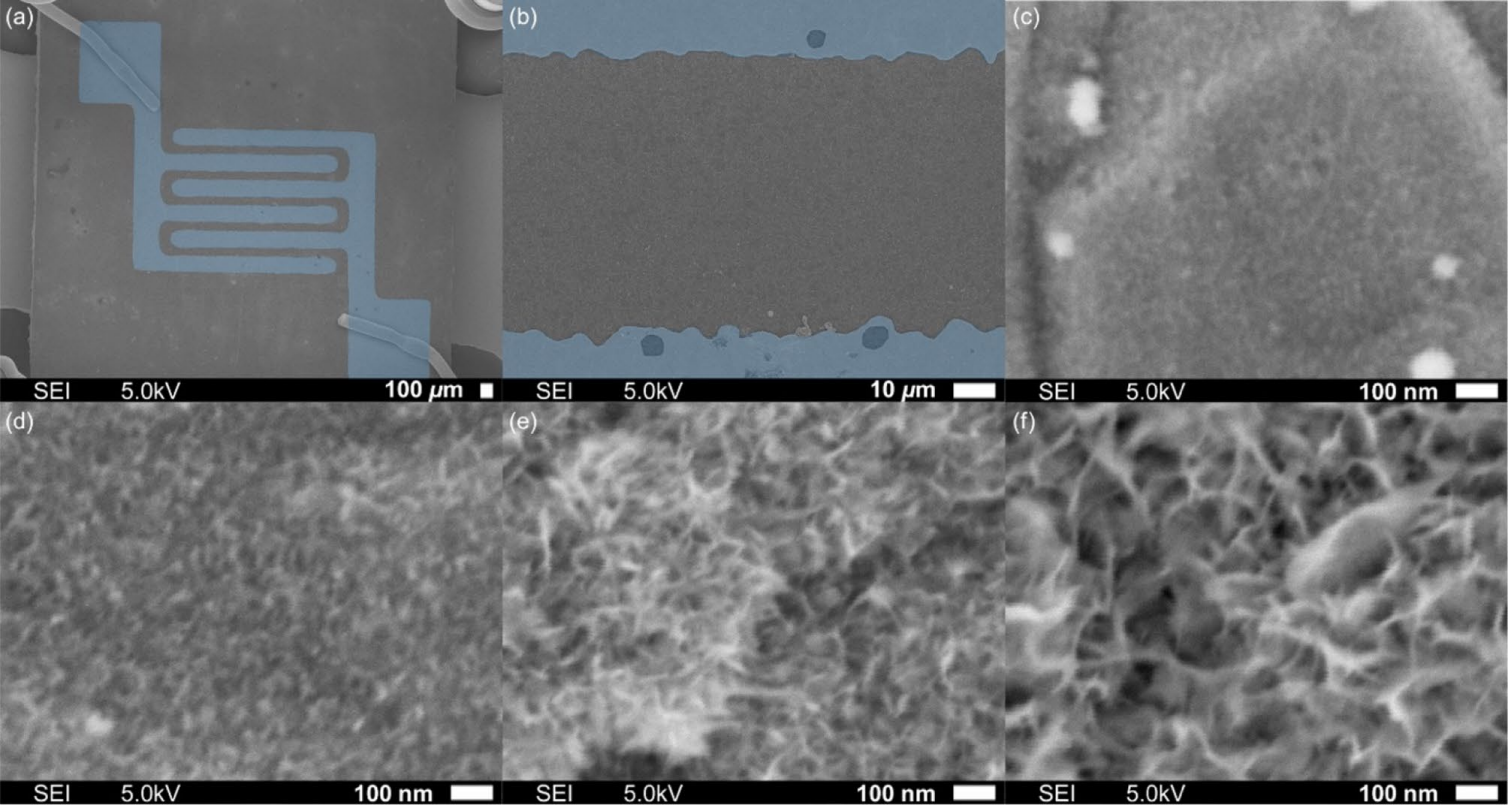
Field emission scanning electron microscopy (FE-SEM) images of the sensor chip (blue area is electrode); (**a**) Pt electrodes on Al_2_O_3_ substrate, (**b**) between Pt electrodes, and nanosheet-type of tin oxide after synthesis for (**c**) 0.5 h, (**d**) 1.0 h, (**e**) 3.0 h, and (**f**) 6.0 h.

Nanosheet-type tin oxide was synthesized on a sensor chip to assess its properties as a gas sensor. Two comb-type electrodes with three digits were well-printed on an Al_2_O_3_ substrate (Fig. 3a), and the distance between the electrodes was approximately 100 μm (Fig. 3b). The nanosheet-type tin oxide was formed in a well-dispersed state on the sensor chip, in which the nanosheet-type tin oxides did not overlap and stand on the substrate, and were rather oriented such that the edge of one was in contact with the flat surface of the other. The size of the flat plane increases with increasing synthesis time (Fig. 3c–f).

**Figure 4:**
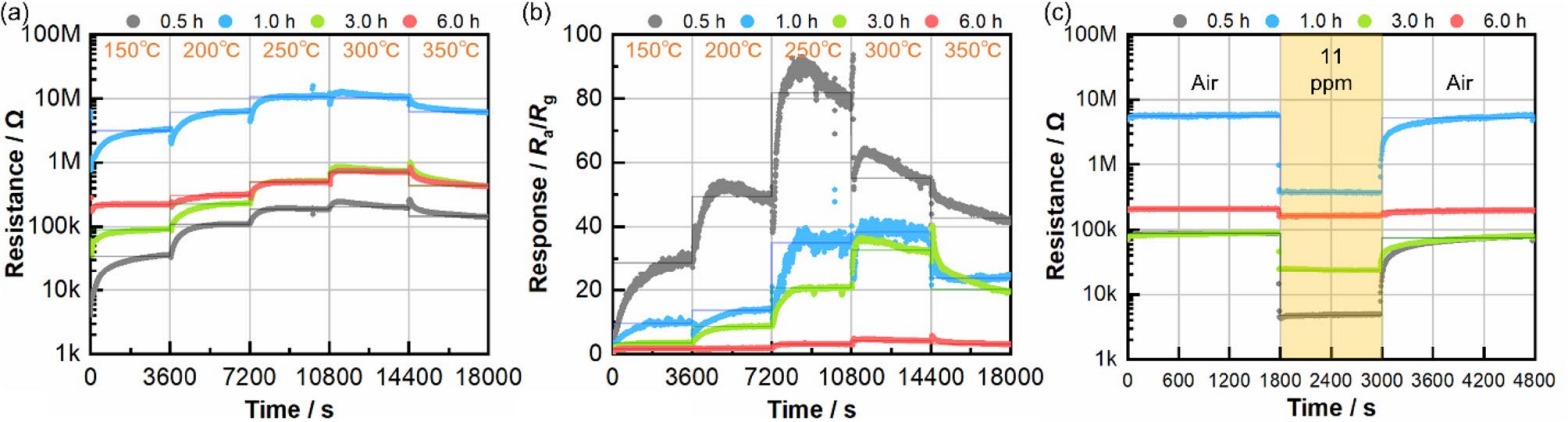
(**a**) Electrical resistance variations under air flow. (**b**) Sensor signal response for 54 ppm allyl mercaptan gas. (**c**) Electrical resistance variation at 300 °C for 11-ppm allyl mercaptan. (Line is the mean value, with less than 1% change in the range.).

The sensor properties were assessed using the as-prepared nanosheet-type tin oxide sensors. A sensor fabricated using commercial tin oxide nanoparticles was also prepared for a comparative study. However, it was difficult to measure the sensor properties owing to their high electrical resistance. In the case of nanosheet-type tin oxide, the electrical resistance was recorded at different temperatures (Fig. 4). Under air flow (Fig. 4a), the electrical resistance increased with increasing temperature from 150 to 300 °C and decreased when the temperature exceeded 300 °C.

The nanosheet-type tin oxide exhibited a fast response to allyl mercaptan (Fig. 4c), which was faster than that of the identical-type of commercial sensors (Fig. S1 and Table S1). The time taken to reach 90% of the response change in the electrical resistance was 5–10 s for a 1200 s flow, and that of the recovery change in the electrical resistance was 800–1100 s for a 1800s flow.

**Figure 5:**
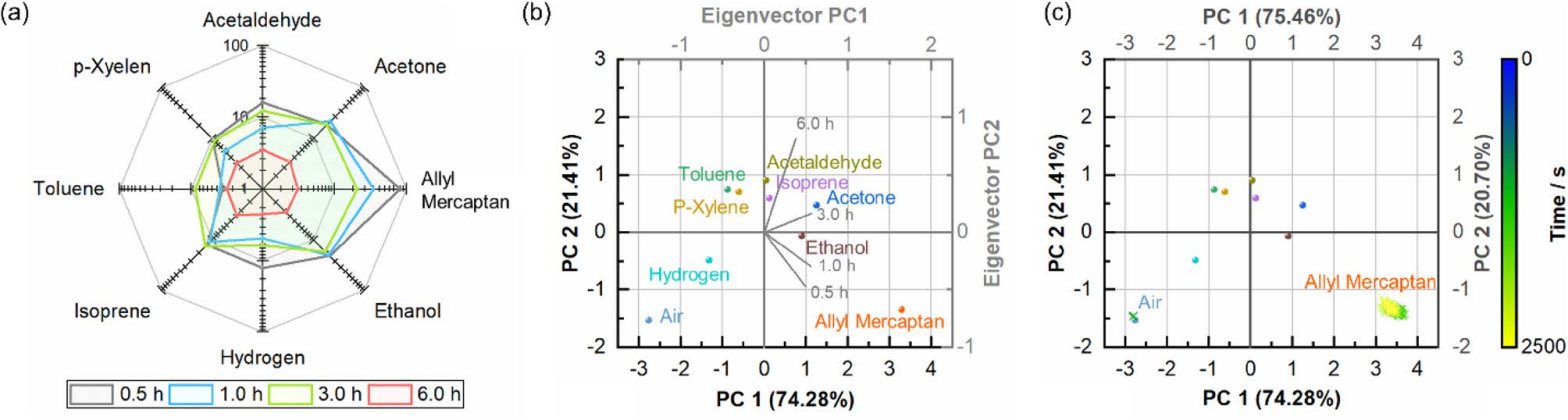
(**a**) Sensor signal response to various gases. (**b**) Scores plot of principal component analysis for various gases (Eigenvector values are Table S3). (**c**) Coordinates plot of air and allyl mercaptan gas on principal component analysis result.

The sensor signal responses to other biomarker gases were also measured for gas discrimination (Figs. S2 and S3). The sensor signal response values are listed in Table S2. Among the measured biomarker gases, nanosheet-type tin oxide exhibited the highest sensor signal response to allyl mercaptan (Fig. 5). The sensor signal response was used for principal component analysis (PCA). Figure 5b shows the PCA scores calculated using the nanosheet-type tin oxide gas-sensor array. The cumulative variance of the first and second principal components was greater than 95%; thus, most of the data were reflected in the PCA results, even after dimensionality reduction. The plotted points were dispersed according to the type of gas used. The data for air was located at the bottom of the third quadrant, whereas that for allyl mercaptan was located at the bottom of the fourth quadrant, with a high degree of separation. In the gas discrimination test, air and allyl mercaptan gas were made to flow into the sensor array. The coordinates for the newly obtained sensor signal response data were obtained and plotted using the pre-calculated PCA results (Fig. 5c). The results showed well-discriminated air and allyl mercaptan gases. The obtained coordinates were initially located near the air area (blue to green marks) and shifted to the allyl mercaptan area over time (green to yellow marks).

**Figure 6:**
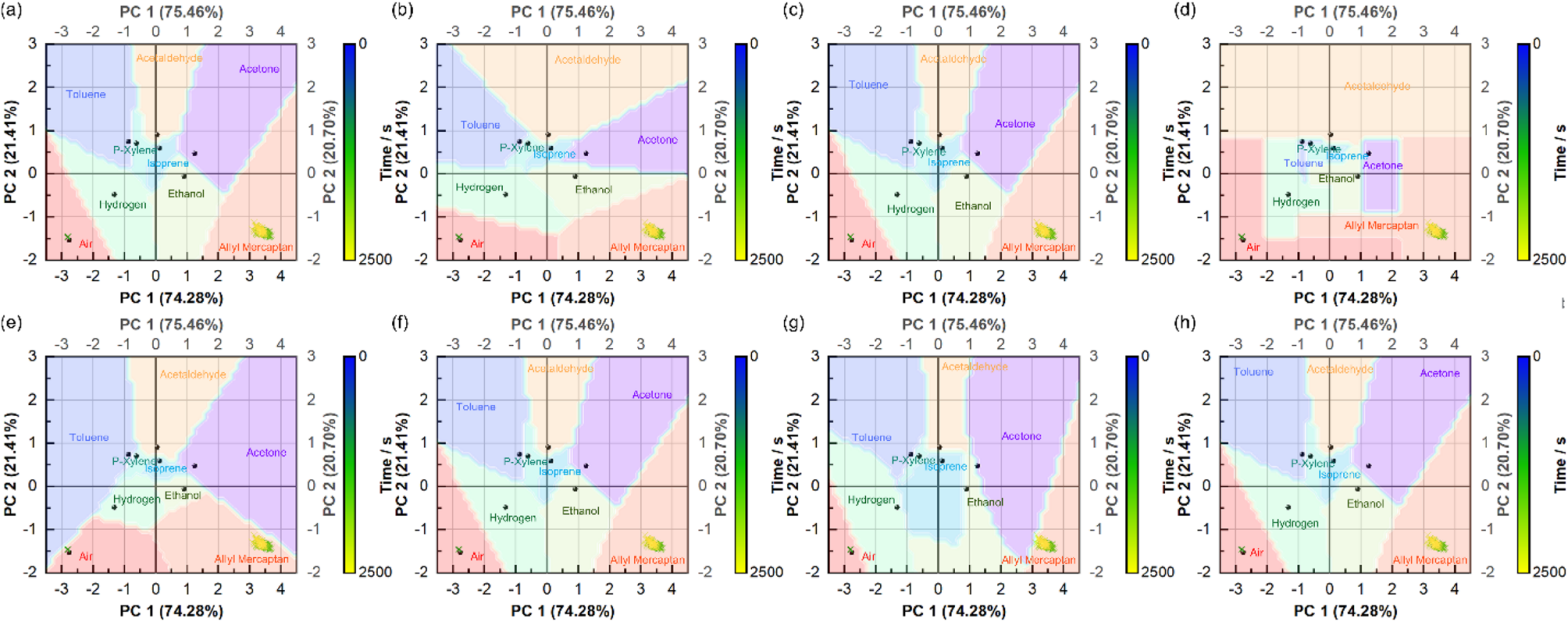
Gas classification models by (**a**) Gaussian naïve Bayes, (**b**) linear discriminant analysis, (**c**) k-nearest neighbor, (**d**) Random forest, (**e**) Linear support vector classification (SVC), (**f**) SVC with linear kernel, (**g**) SVC with polynomial kernel, and (**h**) SVC with radial basis function kernel.

Based on the PCA results for the gas classification, the gas classification models were established using Gaussian naïve Bayes^21,22^, linear discriminant analysis^23–25^, k-nearest neighbor (kNN)^26–29^, random forest^30–32^, and support vector machine (SVM)^33–40^ as supervised learning algorithms. SVMs include models based on linear support vector classification (SVC), SVC with a radial basis function (RBF) kernel, SVC with a polynomial kernel, and SVC linear kernel methods. The classification models are illustrated in Fig. 6. Subsequent verification confirmed 100% classification accuracy with the classification models, except for the SVC with the polynomial kernel classification models, which was 90% for allyl mercaptan (10% was plotted in the boundary between the acetone and allyl mercaptan areas). Comprehensive data from the classification results strongly indicated that the test gases were air and allyl mercaptan.

Nanosheet-type tin oxide exhibited gas concentration dependence for allyl mercaptan (Fig. S4). The sensors exhibited high sensitivity (defined as $\Delta\;sensor\;signal\;response/\Delta\;gas\;concentration$) to allyl mercaptan gas. The highest sensitivity was obtained for 1.0 h synthesized nanosheet-type tin oxide gas sensor. The limit of detection ($LOD=3\times standard\;deviation/sensitivity$) was approximately 200 ppt, which was higher than that of commercial sensors (Table S4). The concentration of allyl mercaptan on the skin was reported in the range of 2.5–12.5 ng/L^41^ and the nanosheet-type tin oxide proed to be a good candidate material for allyl mercaptan gas sensing. Gas classification based on the gas concentration was carried out after adding the gas concentration dependence data in training data. Subsequent verification confirmed 100% classification accuracy with the Gaussian naïve Bayes classification models and over 99% classification accuracy with other classification models (Fig. S5). Less than 1% inaccuracy was noted when the gas flow was changed because of an experimental error caused by mass flow controller.

The electrical resistance variation of the nanosheet-type tin oxide decreased under humid conditions (Fig. S6), indicating a decrease in the sensor signal response. Although the sensor signal response decreased, the nanosheet-type tin oxide showed gas concentration dependence under humid conditions. In addition, nanosheet-type tin oxide showed better sensor properties than commercial gas sensors under humid conditions (Table S5). Gas classification was performed under humid conditions, it was difficult to analyze using the Gaussian naïve Bayes classification models because no contour levels were found within the data range. A classification accuracy of > 99% was obtained for other classification models (Fig. S7).

## Discussion

Nanosheet-type tin oxide shows high catalytic activity toward allyl mercaptan, a known biomarker of mental stress. Nanosheet-type tin oxide was synthesized on the sensor chip via a simple aqueous solution process for use as a gas sensor material to detect allyl mercaptan detection. Under airflow, the electrical resistance increased with increasing temperature from 150 to 300 °C and decreased when the temperature exceeded 300 °C. In general, oxygen on the surface can be present in various chemical states, transferring electrons from tin oxide to oxygen on the surface with changes in temperature, according to the following process:^11^

1 $${\text{O}}_{{2\left( {{\text{gas}}} \right)}} \leftrightarrow {\text{O}}_{{2\left( {{\text{ad}}} \right)}} \leftrightarrow {\text{O}}_{{2\left( {ad} \right)}}^{\prime } \leftrightarrow {\text{O}}_{{\left( {{\text{ad}}} \right)}}^{\prime } \leftrightarrow {\text{O}}_{{\left( {{\text{ad}}} \right)}}^{\prime \prime } \leftrightarrow {\text{O}}_{{2\left( {{\text{lattice}}} \right)}}^{\prime \prime }$$

The increase in the electrical resistance up to 300 °C was related to conversion of the oxygen speciesowing to an increase in the amount of adsorbed oxygen and carrier trapping by adsorbed oxygen, as described above in Eq. (1). Oxygen is known to transfer from ${\text{O}}_{{2\left( {{\text{ad}}} \right)}}^{\prime }$ to ${\text{O}}_{{\left( {{\text{ad}}} \right)}}^{\prime }$ at approximately 150 °C and mainly stabilizes to ${\text{O}}_{{\left( {{\text{ad}}} \right)}}^{\prime \prime }$ at approximately 300 °C^11,42^. The decrease over 300 °C is related to oxygen desorption on the surface, which can be explained as a result of the liberation of trapped electrons from oxygen during desorption. In this study, the electrical resistance under an allyl mercaptan gas flow was measured using an identical method, and the sensor signal response (defined as *R*_a_/*R*_g_, where *R*_a_ and *R*_g_ are the electrical resistances under air and target gas flow, respectively) was calculated (Fig. 4b). The highest sensor signal response to the allyl mercaptan gas was obtained in the temperature range of 250–300 °C. This temperature range was in good agreement with the temperature range that exhibit highest catalytic activity.

There are two responses for the highest sensor signal response temperature range: the adsorption amount of the allyl mercaptan molecule may be at a maximum at 250 °C, and the sensing mechanism. Similary, three types of sensing mechanisms can be considered:^20,43–45^ (i) replacement of the adsorbed oxygen with the target gas molecule, (ii) reaction between the adsorbed oxygen on the surface and a gas molecule, and (iii) reaction between the exposed lattice oxygen and a gas molecule). Cases (i) and (ii) are well known sensing mechanisms for tin oxide gas sensors. When oxygen gets adsorbed onto the surface, it traps electrons from tin oxide, as mentioned in Eq. (1). This electron-trapped state represents the initial stabilized state of the tin oxide gas sensor. When the sensor was exposed to the target gas, the oxygen species replaced and/or reacted with the gas molecules, leading to a decrease in the electrical resistance through the liberation of electrons from oxygen (Eq. 2). The electrical resistance returned to its initial value when the target gas changed to air. A sensor response can be obtained using the variation in the electrical resistance as a signal.

2 $${\text{O}}_{{\left( {{\text{ad}}} \right)}}^{\prime \prime } \leftrightarrow \frac{1}{2}{\text{O}}_{{2\left( {{\text{gas}}} \right)}} + 2e^{\prime}$$

In this study, the predominant oxygen species on the (110) crystal facets of the tin oxide nanoparticles was chemisorbed oxygen (approximately 91%), whereas the oxygen species on the (101) crystal facets of the nanosheet-type tin oxide were found to be Sn bound oxygen in the lattice (approximately 48%), oxygen attributed to crystal defects (approximately 30%), and chemisorbed oxygen (approximately 22%). This indicates that case (iii) can become the predominant reaction on nanosheet-type tin oxide. In the case of nanosheet-type tin oxide, the gas molecules react with the lattice oxygen, thereby liberating electrons and forming oxygen vacancies (Eq. 3). Lattice oxygenserves as bridging oxygen^15,46^

3 $${\text{O}}_{{\text{o}}}^{ \times } \leftrightarrow \frac{1}{2}{\text{O}}_{{2\left( {{\text{gas}}} \right)}} + V^{ \cdot \cdot } + 2e^{\prime}$$

The fabricated gas sensors exhibited a high gas selectivity for allyl mercaptan; therefore, a sensor array consisting of nanosheet-type tin oxide was prepared. Nanosheet-type tin oxides synthesized for 0.5, 1.0, 3.0, and 6.0 h were used as the sensor array. Data from the sensor array were collected using biomarker gases, such as allyl mercaptan, acetone, acetaldehyde, ethanol, hydrogen, isoprene, toluene, and p-xylene. PCA was conducted using the sensor signal response from the sensor array, and the allyl mercaptan data were located at the bottom of the fourth quadrant, with a high degree of separation. Gas classification models were built using a supervised learning algorithm based on PCA results using the sensor signal response from the sensor array. Although the sensing properties decreased slightly under humid conditions, the comprehensive data from the classification models were forecasted as allyl mercaptan with high accuracy. The nanosheet-type tin oxide is a key material for improving the forecasting accuracy of mental stress monitoring.

## Methods

Nanosheet-type tin oxide was synthesized using SnF_2_ (90.0% pure, FUJIFILM Wako Pure Chemical Corp.) via an aqueous solution. An aqueous solution of SnF_2_ (0.028 M) was prepared and maintained at 90 °C for 6 h. The precipitate was collected and dried at room temperature. Commercial tin oxides (nanopowder, ≤ 100 nm avg. part. size; Sigma-Aldrich Co., LLC) was used for comparison. The allyl mercaptan gas was generated using allyl mercaptan (> 70%, Tokyo Chemical Industry Co., Ltd.) via a gas generator (PD-1B, GASTEC Co.) The allyl mercaptan oxidation activity was assessed in a fixed bed flow reactor consisting of a quartz glass tube (diameter: 10 mm) under a 100 cm^3^/min flow of 54-ppm allyl mercaptan gas over 0.01 g of tin powder. A nanosheet-type tin oxide gas sensor was fabricated using similar process. The platinum electrode printed aluminum oxide sensor chip was cleaned by UV light irradiation light (PL16-10 low-pressure mercury lamp, air flow, 100 V, 200 W, SEN Lights Co.) for 20 min. to ensure effective nucleation and growth of nanosheet-type tin oxide. Subsequently the cleaned sensor chip was immersed in an aqueous solution of SnF_2_ at 90 °C for 0.5, 1, 3, or 6 h. The sample was washed under running water, followed by blow drying at room temperature. The morphology and structure were observed using TEM (Tecnai Osiris, FEI) and FE-SEM (JSM-6335FM, JEOL Ltd.). The gas sensing properties were assessed using a gas sensor evaluation module. A mixed gas (99.99995% nitrogen:99.99995% oxygen = 80:20) was used as air gas. The concentration of allyl mercaptan was controlled by mixing it with air gas, where the gas flow was set to 100, 150, 200, 300, 400, and 500 cm^3^/min for 54, 36, 27, 18, 14, and 11 ppm, respectively. The concentrations of acetaldehyde, acetone, ethanol, hydrogen, isoprene, toluene, and p-xylene were set to 20 ppm and 100 cm^3^/min by mixing N_2_-balanced 25 ppm gas with 99.9995% N_2_ gas. The humidity effect was examined using a typical nitrogen–oxygen balanced gas-bubbler system. Commercial gas sensors (TGS2600, TGS2602, TGS2603, TGS2610C0, TGS2610D0, TGS2611C0, TGS2611E0, TGS2612D0; FIGARO) were simultaneously used for comparison. The hyperparameters for the depth and random state in the random forest model were 2 and 9, respectively. The k value in the kNN model was set to 9. The gamma value in the SVC with RBF kernel was 0.7. The degree value in the SVC with polynomial SVC was 9. The other hyperparameters were automatically set.

## Supplementary Information

Supplementary Information.

## Data Availability

All data generated or analysed during this study are included in this published article and its supplementary information files.
