# Supplementary material for: Nanosheet-type tin oxide gas sensor array for mental stress monitoring
Source: Sci Rep. 2022 Aug 25;12:13874. doi: 10.1038/s41598-022-18117-8 (PMC9411192; doi:10.1038/s41598-022-18117-8)
Supplement: Supplementary file 1 — Supplementary Information. [file 41598_2022_18117_MOESM1_ESM.pdf]

Supplementary material

Nanosheet-Type Tin Oxide Gas Sensor Array for Mental Stress Monitoring

Pil Gyu Choi[1]*, and Yoshitake Masuda[1]

[1]National Institute of Advanced Industrial Science and Technology (AIST), 2266-98 Anagahora, Shimoshidami, Moriyama, Nagoya, 463-8560, Japan

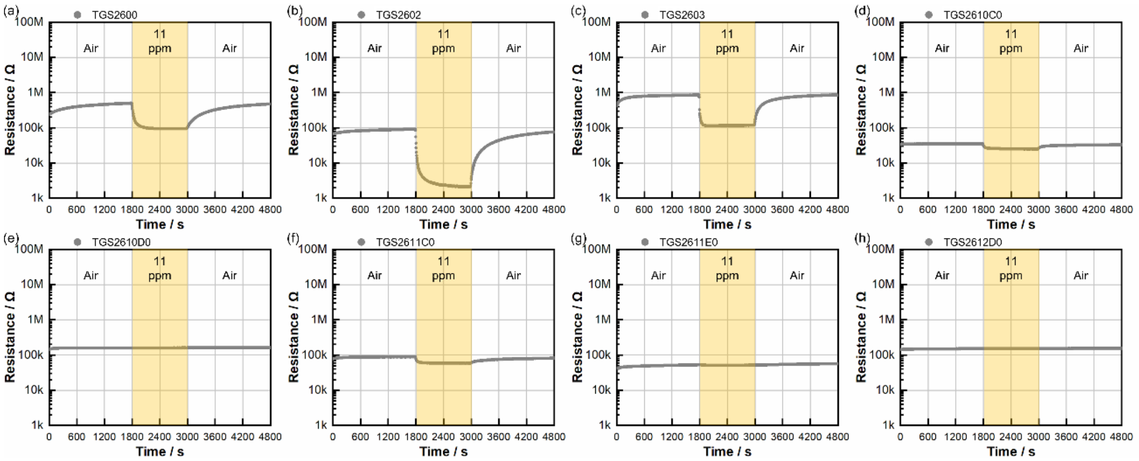

Figure S1. Electrical resistance variation for 11-ppm allyl mercaptan.

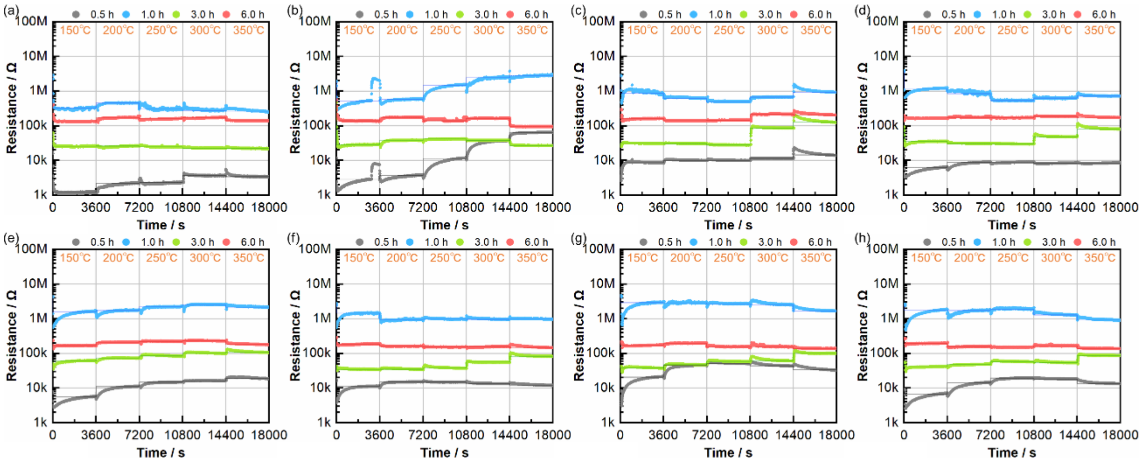

Figure S2. Electrical resistance variations under 100 cm^3 min^{-1} flow of (a) 54 ppm allyl mercaptan, (b) 20 ppm acetaldehyde, (c)20 ppm acetone, (d) 20 ppm ethanol, (e) 20 ppm hydrogen, (f) 20 ppm isoprene, (g) 20 ppm toluene, and (h) 20 ppm p-xylene.

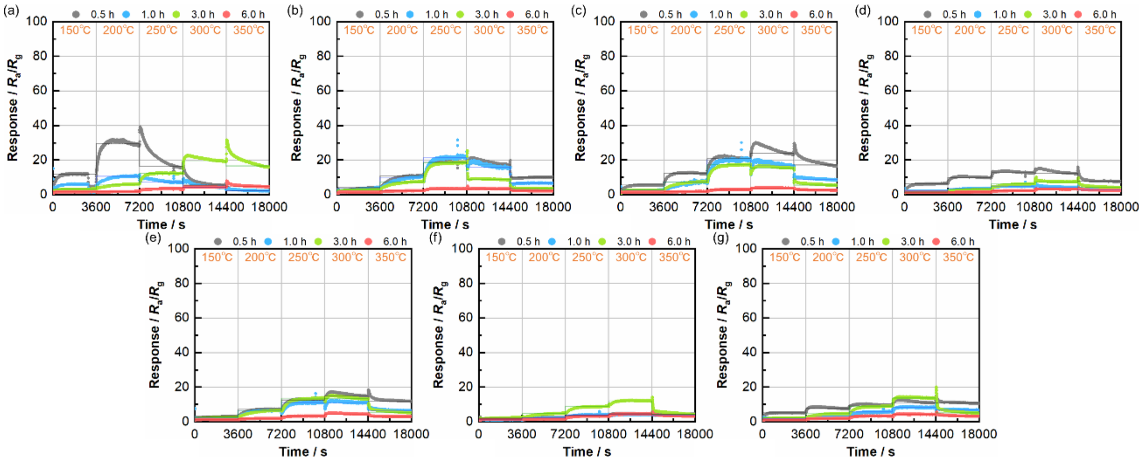

Figure S3. Sensor signal response for 100 cm^3 min^{-1} flow of (a) 20 ppm acetaldehyde, (b)20 ppm acetone, (c) 20 ppm ethanol, (d) 20 ppm hydrogen, (e) 20 ppm isoprene, (f) 20 ppm toluene, and (g) 20 ppm p-xylene.

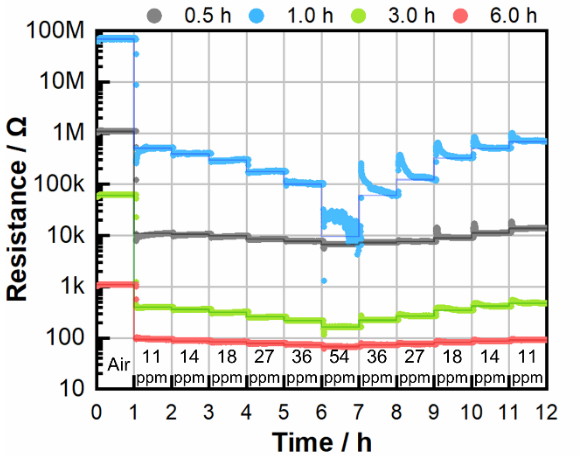

Figure S4. Electrical resistance variations under air and different concentrations of allyl mercaptan flow.

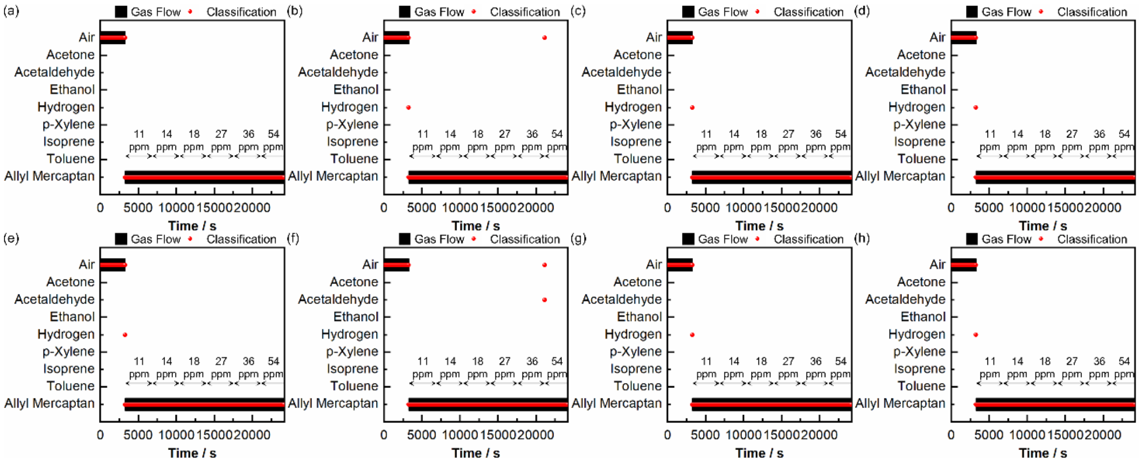

Figure S5. Gas flow and classification by (a) Gaussian naïve Bayes, (b) linear discriminant analysis, (c) k-nearest neighbor, (d) Random forest, (e) Linear support vector classification (SVC), (f) SVC with linear kernel, (g) SVC with polynomial kernel, and (h) SVC with radial basis function kernel.

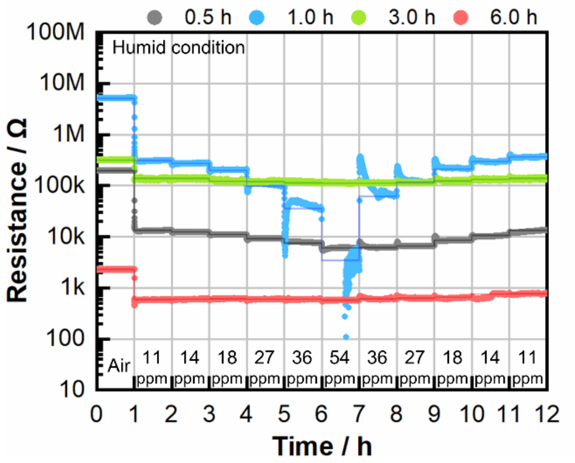

Figure S6. Electrical resistance variations under air and various concentration of allyl mercaptan flow under humid condition.

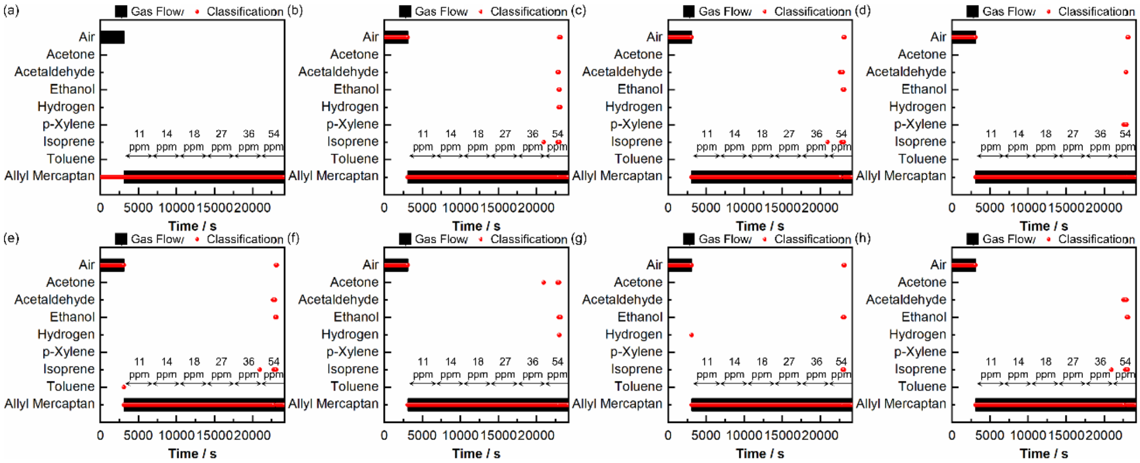

Figure S7. Gas flow and classification under humid condition by (a) Gaussian naïve Bayes, (b) linear discriminant analysis, (c) k-nearest neighbor, (d) Random forest, (e) Linear support vector classification (SVC), (f) SVC with linear kernel, (g) SVC with polynomial kernel, and (h) SVC with radial basis function kernel.

Table S1. Response time and recovery time for 11-ppm allyl mercaptan gas

Gas sensor	Condition	90% response time / s	90% recovery time / s
0.5h	300°C	5	1085
1.0h	300°C	5	905
3.0h	300°C	10	1080
6.0h	300°C	10	825
TGS2600	5V	100	1305
TGS2602	5V	40	1365
TGS2603	5V	25	1030
TGS2610C0	5V	190	660
TGS2610D0	5V	-	-
TGS2611C0	5V	120	1070
TGS2611E0	5V	-	-
TGS2612D0	5V	-	-

Table S2. 3600-s flow sensor signal response to gases

Gas	Flow / $cm^3 \cdot min^{-1}$	Temperature/ °C	Sensor signal response / R_a/R_g			
			0.5 h	1.0 h	3.0 h	6.0 h
54-ppm Allyl mercaptan	100	150	28.58	9.68	3.48	1.66
		200	49.34	13.66	8.64	1.77
		250	81.75	35.00	20.68	3.10
		300	55.23	38.32	32.56	4.08
		350	42.56	23.67	20.18	3.11
20-ppm Acetaldehyde	100	150	11.83	5.96	3.14	1.58
		200	29.35	10.58	5.91	1.75
		250	16.36	7.16	12.25	3.56
		300	5.48	4.29	19.54	4.28
		350	2.27	2.18	16.57	4.62
20-ppm Acetone	100	150	3.99	3.59	2.89	1.36
		200	10.84	9.93	7.36	2.17
		250	18.63	21.49	18.33	3.39
		300	17.56	15.71	8.65	3.26
		350	10.06	6.59	3.44	2.10

20-ppm Ethanol	100	150	5.59	2.66	2.54	1.32
		200	12.28	7.34	7.30	1.74
		250	20.76	20.12	17.19	2.90
		300	23.93	16.70	15.49	3.75
		350	17.10	8.65	5.48	2.52
20-ppm Hydrogen	100	150	6.25	1.99	1.46	1.31
		200	9.86	3.55	3.09	1.46
		250	12.65	4.88	6.08	2.22
		300	12.35	4.13	7.38	3.07
		350	7.63	2.87	4.13	2.43
20-ppm Isoprene	100	150	3.05	2.19	2.58	1.17
		200	7.23	6.49	6.46	1.90
		250	12.61	11.07	13.54	3.25
		300	15.03	11.00	13.31	4.45
		350	11.95	6.37	5.39	2.96
20-ppm Toluene	100	150	1.67	1.08	2.35	1.27
		200	2.42	2.14	4.77	1.55
		250	3.65	3.96	8.75	3.16

		300	4.57	4.12	12.11	4.50
		350	4.30	3.69	4.39	3.17
20-ppm p-Xylene	100	150	5.11	1.81	2.14	1.12
		200	7.63	3.55	4.67	1.94
		250	9.60	5.61	8.86	3.28
		300	11.00	8.18	13.70	4.22
		350	10.72	6.76	5.10	3.17

Table S3. Eigenvector values of the covariance of sensor signal response dataset at 250 °C

Sensor	Eigenvector PC1	Eigenvector PC2
0.5 h	0.49460	-0.47416
1.0 h	0.55104	-0.29116
3.0 h	0.56048	0.16582
6.0 h	0.37094	0.81419

Table S4. Sensitivity and LOD to allyl mercaptan

Gas sensor	Sensitivity	Standard deviation	LOD (Limit of detection) / ppt
0.5h	4.0157	2.35×10^{-4}	176
1.0h	81.738	6.02×10^{-3}	221
3.0h	8.0138	5.05×10^{-4}	189
6.0h	0.3965	2.72×10^{-4}	2057
TGS2600	1.6838	5.91×10^{-3}	105244
TGS2602	1.4427	4.20×10^{-4}	873
TGS2603	1.4792	6.22×10^{-4}	832
TGS2610C0	0.0205	5.22×10^{-4}	763453
TGS2610D0	0.0032	1.49×10^{-4}	1399433
TGS2611C0	0.0781	7.82×10^{-3}	3002247
TGS2611E0	0.0636	3.24×10^{-4}	152800
TGS2612D0	0.0037	2.10×10^{-4}	1702089

$sensitivity = \Delta sensor\ signal\ response/\Delta gas\ concentration$

$LOD = 3 \times standard\ deviation/sensitivity$

Table S5. Sensitivity and LOD to allyl mercaptan under humid condition

Gas sensor	Sensitivity	Standard deviation	LOD (Limit of detection) / ppt
0.5h	0.8002	7.84×10^{-5}	2938
1.0h	15.993	6.41×10^{-3}	1202
3.0h	0.0490	1.90×10^{-2}	1165232
6.0h	0.0829	4.99×10^{-3}	180566
TGS2600	0.1367	1.55×10^{-3}	34000
TGS2602	0.5482	1.64×10^{-3}	8980
TGS2603	0.2332	1.06×10^{-3}	13648
TGS2610C0	0.0058	2.76×10^{-4}	142746
TGS2610D0	0.0035	2.29×10^{-4}	195833
TGS2611C0	0.0211	3.34×10^{-3}	476083
TGS2611E0	-	-	-
TGS2612D0	0.0050	3.08×10^{-4}	184774

$$sensitivity = \Delta sensor\ signal\ response/\Delta gas\ concentration$$

$$LOD = 3 \times standard\ deviation/sensitivity$$
